# Meniscus Morphology-Based Prediction of Backup Roll Eccentricity for Stable Slot-Die Coating on Polymer Films

**DOI:** 10.3390/polym18162007

**Published:** 2026-08-17

**Authors:** Mingi Kim, Chanwoo Kim, Jeongdai Jo, Byungho Park, Changwoo Lee

**Affiliations:** 1 Department of Mechanical Design and Production Engineering, Konkuk University, 120 Neungdong-ro, Seoul 05029, Republic of Korea; gxkr508@konkuk.ac.kr (M.K.); kcw9817@konkuk.ac.kr (C.K.); 2 Department of Advanced Battery Manufacturing Systems, Korea Institute of Machinery and Materials, 156 Gajeongbuk-ro, Daejeon 34103, Republic of Korea; micro@kimm.re.kr; 3 Flexible Production Systems Research Department, Korea Textile Machinery Convergence Research Institute, 27 Sampung-ro, Gyeongsan-si 38542, Republic of Korea; bhpark@kotmi.re.kr; 4 Department of Mechanical Engineering, Konkuk University, 120 Neungdong-ro, Seoul 05029, Republic of Korea

**Keywords:** meniscus morphology, polymer film, roll eccentricity, slot-die coating, vision data

## Abstract

In roll-to-roll slot-die coating systems, backup roll eccentricity induces periodic fluctuations in key process parameters, leading to coating defects. Therefore, accurate prediction of backup roll eccentricity during operation is essential for maintaining stable coating quality. However, conventional methods based on web tension signals have limitations in clearly distinguishing and quantitatively evaluating subtle eccentricities. To address this issue, this study proposes a data-driven framework for predicting backup roll eccentricity using meniscus image information. Representative morphological features are defined to describe the global shape, local shape, and curvature characteristics of the meniscus. Since these features are sensitive to eccentricity-induced process variations, they can serve as effective indicators for eccentricity prediction. The defined features are used to train regression models, and the model with the highest predictive accuracy is selected. Experimental results confirm that the proposed meniscus-based approach significantly outperforms conventional tension-based methods. The proposed method achieves an average Root Mean Square Error (RMSE) of approximately 0.63 μm, an average Normalized Root Mean Square Error (NRMSE) of approximately 0.34, and a coefficient of determination (R^2^) greater than 0.93 across all test cases. These results demonstrate the feasibility of robust process monitoring using a simple vision sensor configuration.

## 1. Introduction

Roll-to-roll (R2R) slot-die coating systems are core manufacturing processes used for forming uniform coating layers on continuously transported flexible substrates [1,2,3]. These systems have been widely employed in various industrial applications, which include lithium-ion batteries, solar cells, and solid oxide fuel cell electrolytes because of their capability for large-area and high-throughput deposition of functional thin films [4,5,6,7,8]. The R2R process includes unwinding, coating, drying, and rewinding stages, wherein the web tension and transport speed are precisely regulated by driven rolls within each span [9,10,11]. Slot-die coating operates in a pre-metered manner, wherein the coating layer thickness is determined by prescribed process parameters [12,13]. Therefore, maintaining key variables-such as web tension, web speed, and coating gap within stable operating ranges is essential for achieving target thickness and coating uniformity [14]. These process variables directly affect the hydrodynamic behavior of the meniscus formed between the coater lip and moving web. Variations in the meniscus position and shape arise when disturbances occur during operation, often leading to coating defects such as machine direction (MD) thickness deviation, streaks, and ribbing [15,16,17]. This sensitivity becomes critical in processes producing ultrathin coating layers with thicknesses of only a few micrometers or less, where even minor disturbances can result in severe degradation of the coating quality [18]. Among various disturbance sources, roll eccentricity is a common and significant mechanical defect in slot-die coating processes [19]. Roll eccentricity originates from initial manufacturing imperfections, long-term surface wear, or bearing degradation during operation [20,21]. When eccentricity is present, the effective roll radius varies periodically with the rotational angle, inducing synchronous periodic fluctuations in key process variables such as web tension and coating gap. Even a subtle eccentricity in the backup roll, which directly supports the web beneath the slot-die, can destabilize the meniscus flow and cause severe coating defects. Previous studies relied on tension signal analysis combined with system dynamic models that account for web tension transmission characteristics to predict roll eccentricity. Yun et al. [22] analyzed periodic tension fluctuations induced by roll eccentricity in R2R systems and proposed a data-driven approach that predicts eccentric roll conditions using statistical features extracted from circumferentially segmented tension signals. Lee et al. [23] developed a dynamic system model by integrating rolling force models with tension transmission characteristics based on strip thickness and tension measurements, which enables the reconstruction of roll radius variation along the width direction and the prediction of three-dimensional roll shape defects involving periodic eccentricity. Seki et al. [24] investigated the generation mechanism of periodic disturbance components embedded in tension responses using a dynamic model of R2R web handling systems, demonstrating that roll eccentricity can be identified by detecting tension components synchronized with the roll rotational frequency. In addition, Lee et al. [19] analyzed tension amplification characteristics associated with wrap angle and tension sensor placement and proposed a framework that simultaneously predicts the presence and location of roll eccentricity using a feature subset selection method combined with a support vector machine. Although these approaches are effective for identifying relatively large eccentricities or eccentricities in driven rolls, they exhibit inherent limitations in precisely detecting localized or subtle eccentricities. Tension signals reflect the overall dynamic behavior of an entire span, and therefore, the local eccentricity effects are often obscured. In practical processes, spatial nonuniformity in web thickness and material properties can induce localized variations in the tension distribution within a span. Further, the tension signals are simultaneously affected by multiple factors, including drive-speed mismatch, acceleration and deceleration conditions, and external mechanical disturbances [25]. Consequently, it becomes difficult to isolate eccentricity-induced tension components from other disturbance sources. This limitation is pronounced for small-magnitude eccentricity occurring in non-driven rolls such as backup rolls, where the signal-to-noise ratio (SNR) of the eccentricity-related component in the tension signal is inherently low.

To overcome these limitations, this study proposes an eccentricity prediction framework based on meniscus image data. The meniscus shape exhibits a strong sensitivity to variations in process conditions caused by its inherent fluid dynamic characteristics, which makes it a valuable indicator to evaluate coating process states. For example, Kim et al. [14] demonstrated that coating-layer thickness can be quantitatively predicted using machine-learning models trained on meniscus images acquired during slot-die coating processes. Similarly, Na et al. [26] reported that the real-time monitoring of the meniscus shape enables the early detection of dead-zone formation and reveals correlations between meniscus behavior and process parameter variations, confirming the potential of meniscus geometry as an early indicator of coating defects. Despite these advances, a quantitative estimation of backup roll eccentricity based on meniscus shape analysis is yet to be reported.

In this study, a data-driven model is developed to precisely predict roll eccentricity by quantitatively analyzing meniscus image data. To this end, a set of morphological features is extracted from meniscus images acquired during slot-die coating operations. The corresponding meniscus shape variations induced by process disturbances were analyzed systematically. Key features exhibiting a strong correlation with eccentricity were selected to construct a regression-based prediction model, which enables the quantitative estimation of subtle eccentricities difficult to predict using conventional tension-based approaches.

## 2. Theoretical Background

### 2.1. Problem Statement

**Figure 1:**
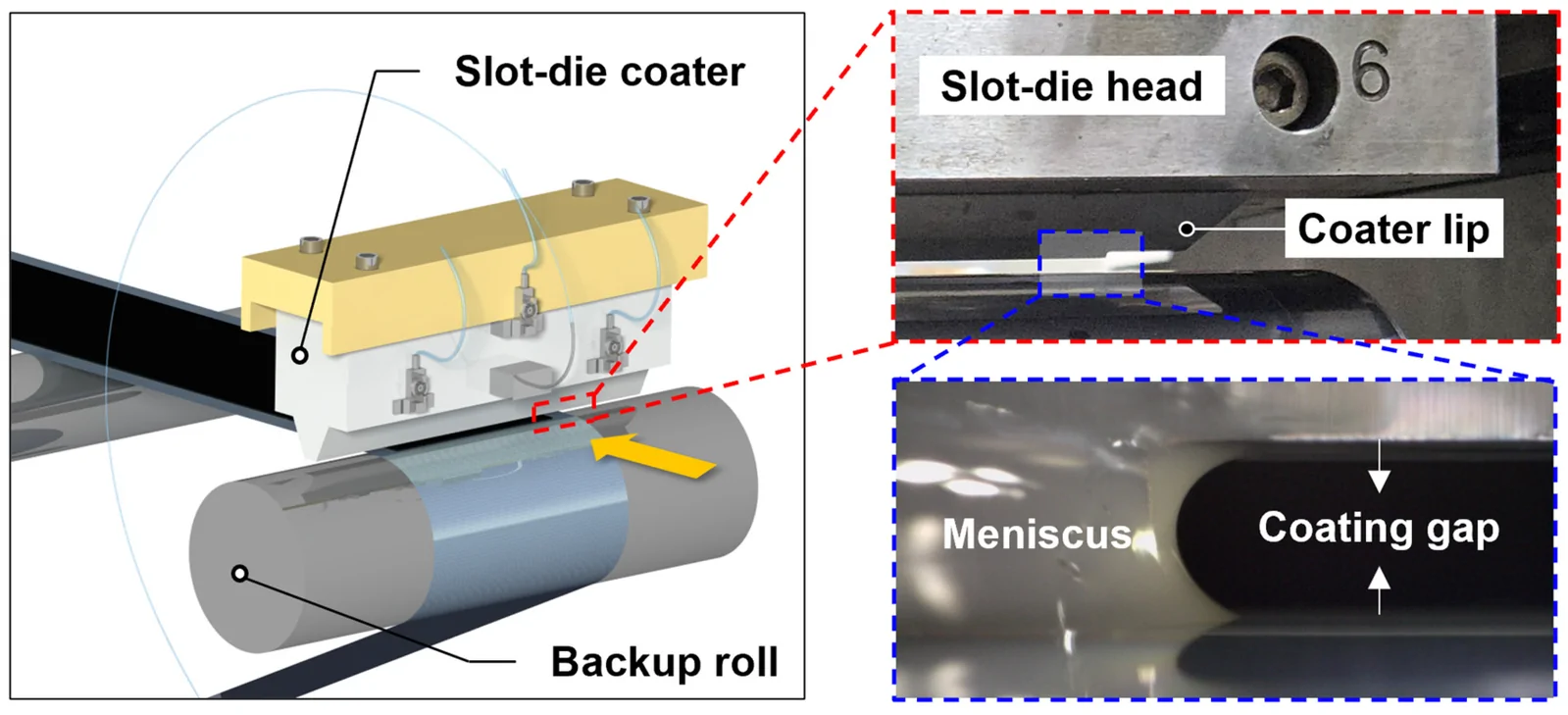
Schematic of a roll-to-roll slot-die coating system, with an enlarged MD view of meniscus formation.

Slot-die coating is a representative noncontact continuous coating process that enables the precise control of the coating thickness on thin and flexible films, commonly referred to as webs [27,28,29,30]. As illustrated in Figure 1, in an R2R slot-die coating system, the slot-die coater is positioned above the moving web, and the web is transported at a constant speed along a backup roll. A predefined gap referred to as the coating gap is maintained between the coater lip and web surface. The coating liquid is supplied at a controlled flow rate to the slot-die head through an external delivery system and continuously discharged through the coater lip onto the moving web surface. During this process, the discharged coating liquid forms a curved free surface known as a meniscus within the coating gap because of the balance between surface tension and viscous forces. This meniscus region includes a critical flow zone that governs the stability of the coating bead and directly affects the final coating layer thickness [31]. In practical slot-die coating operations, process variables are subject to temporal fluctuations caused by the mechanical imperfections of equipment and variations in operating conditions [32,33]. When geometric errors or eccentricity are present in the backup roll, key process variables such as web tension, web speed, and coating gap exhibit periodic fluctuations synchronized with roll rotation. Even small-amplitude variations in these process variables can disrupt the force balance within the meniscus, inducing changes in its shape and position. Subsequently, such meniscus deformation can result in nonuniform coating thickness and various coating defects [34]. Therefore, accurately predicting the backup roll eccentricity, one of the primary sources of meniscus instability and process-induced disturbances, is essential for the stable operation of slot-die coating processes.

### 2.2. Periodic Variation of Process Variables Induced by Roll Eccentricity

**Figure 2:**
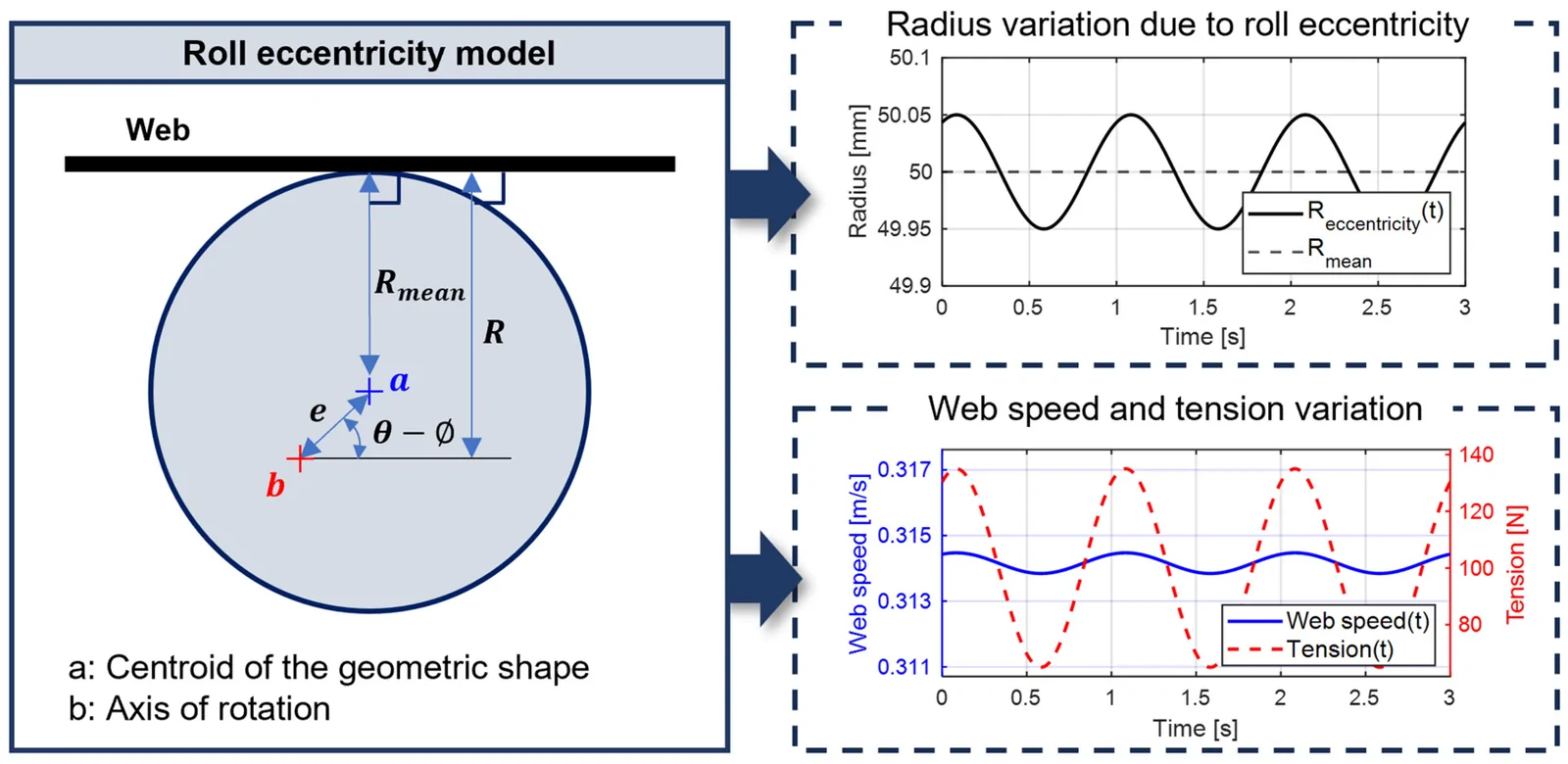
Example of effective roll radius variation caused by roll eccentricity.

In an ideal slot-die coating system, a precisely manufactured backup roll maintains a constant radius regardless of its rotational angle. However, the contact position supporting the web varies periodically with the roll rotation when eccentricity arises because of surface wear or geometric deformation of the roll. Figure 2 illustrates the resulting periodic variation in the effective roll radius induced by eccentricity. Accordingly, the effective radius supporting the web can be expressed as a function of the rotational angle.

(1) $$R\left(\theta \right)=R_{mean}+ecos\left(\theta -\emptyset \right),$$

where R(θ), $R_{mean}$, e, θ, and ∅ represent the effective radius, mean radius, magnitude of the eccentricity, rotational angle, and phase angle of the eccentricity, respectively. Thus, the effective radius periodically increases and then decreases when the roller completes one full revolution. Such variations in the effective radius directly affect the coating gap. The coating gap reaches its minimum value when the position of maximum eccentricity passes directly beneath the coater lip. Further, variations in the effective radius change the tangential surface velocity of the roll and directly affect the web transport speed. The web transport speed is expressed using

(2) $$V_{web}\left(\theta \right)=\omega R\left(\theta \right),$$

where $V_{web}\left(\theta \right)$ and ω represent the web transport speed and angular velocity of the roll, respectively. In the presence of eccentricity, the effective radius varies according to Equation (1). Consequently, the web speed fluctuates periodically. In this case, the fluctuation in web speed generates a speed deviation relative to the set speed $V_{0}$, which is defined by

(3) $$\Delta V\left(\theta \right)=V_{web}\left(\theta \right)-V_{0}=\omega \left(R_{mean}+ecos\left(\theta -\emptyset \right)\right)-\omega R_{mean}=\omega ecos\left(\theta -\emptyset \right).$$

In an R2R system, the web tension is generated by the speed difference between two adjacent driven rolls within a span [35]. Under steady-state conditions, the speed difference and resulting web tension remain constant. However, when a roll with eccentricity is located within a span, the local web transport speed at that position fluctuates with the roll rotation. The resulting speed deviation induces deformation of the web within the span, leading to dynamic fluctuations in web tension [36]. Assuming a linear elastic model for the web, the variation in tension can be expressed as

(4) $$\Delta T\left(\theta \right)=EA\frac{\Delta V\left(\theta \right)}{V_{0}},$$

where ΔT(θ), E, and A represent the tension variation, elastic modulus, and cross-sectional area of the web, respectively. By substituting Equation (3) into Equation (4), the tension fluctuation induced by eccentricity can be expressed as

(5) $$\Delta T\left(\theta \right)=\frac{EAe}{R_{mean}}\cos\left(\theta -\emptyset \right).$$

According to this model, when the material properties remain constant, periodic tension fluctuations occur depending on the magnitude and phase of eccentricity. Such periodic variations in process variables are not limited to mechanical instability but also directly affect the shape of the meniscus formed between the slot-die coater and the web.

### 2.3. Correlation Between Meniscus Morphology and Roll Eccentricity

In the slot-die coating process, the meniscus formed between the coater lip and moving web is maintained by balancing capillary forces and viscous forces within the narrow region [37,38]. The force balance changes when variations in process conditions occur, and the meniscus shape varies accordingly. The effective radius of the roll varies periodically with the rotational angle when roll eccentricity is present.

Consequently, major process parameters such as the web tension, web speed, and coating gaps fluctuate periodically. Such mechanical disturbances alter the force balance associated with flow conditions, and accordingly, the meniscus shape varies dynamically. The mechanical flattening of the web reduces surface roughness with an increase in web tension, thereby leading to an increase in the effective surface energy [39]. These changes in surface characteristics modify contact conditions between the liquid and solid surfaces, thereby resulting in a decrease in the contact angle. Consequently, the wettability is enhanced, and the meniscus shape is altered. This relationship can be described by the Young–Dupré equation [40], which is expressed as

(6) $$W_{SL}=\gamma_{L}\left(1+\cos\theta_{Y}\right),$$

where $W_{SL}$, $\theta_{Y}$, and $\gamma_{L}$ represent the work of adhesion, Young’s contact angle, and surface tension at the liquid–vapor interface, respectively. Assuming no energy transfer across the interfaces, periodic fluctuations in tension lead to variations in the surface contact angle of the web, thereby resulting in corresponding variations in the meniscus shape. Meanwhile, the capillary number (Ca), representing the ratio of viscous forces to surface-tension forces increased with increasing web speed. This relationship is defined by

(7) $$Ca=\frac{\mu V}{\gamma_{L}},$$

where μ represents the viscosity of the coating liquid. The pressure distributions upstream and downstream of the meniscus changed with an increase in Ca; consequently, the curvature of the meniscus increased. That is, the meniscus deformed into a steeper and shorter shape with an increase in web speed. In addition, the flow channel between the coater lip and web narrows locally when the coating gap is temporarily reduced because of roll eccentricity. Viscous flow within a thin flow channel can be approximated using the Reynolds equation when the coating gap is sufficiently small [41]. This is expressed by

(8) $$\frac{dP}{dx}=\frac{12\mu U}{h^{2}},$$

where P, U, and h represent the local pressure, mean flow velocity, and coating gap, respectively. As indicated in Equation (8), the pressure gradient increased rapidly with a decrease in coating gap. Consequently, the pressure difference within the coating bead increased even at the same flow velocity. An increase in pressure difference induces changes in capillary pressure between the interior and exterior of the meniscus [42]. This can be explained by the Young–Laplace equation expressed as

(9) $$\Delta P=\gamma \left(\frac{1}{R_{1}}+\frac{1}{R_{2}}\right),$$

where $R_{1}$ and $R_{2}$ are the radii of curvature in the flow and width directions, respectively. The radius of curvature decreases with an increase in pressure difference; consequently, this increases the curvature of the meniscus. Such physical mechanisms confirm that the meniscus shape is closely correlated with process parameter variations induced by roll eccentricity. However, in practical slot-die coating processes, multiple process variables, including web tension, web speed, and coating gap, act simultaneously, which results in nonlinear variations in the meniscus shape. Consequently, it is difficult to quantitatively derive roll eccentricity solely from meniscus shape variations using conventional mathematical models.

## 3. Proposed Method

In R2R slot-die coating systems, the meniscus shape exhibits high sensitivity to subtle variations in process conditions such as web tension, web speed, and coating gap. Owing to this sensitivity, the meniscus morphology can effectively reflect process state changes, which include small-magnitude roll eccentricity effects. This is often overlooked by tension-based signals; however, the resulting meniscus shape variations are highly nonlinear and cannot be quantitatively characterized using a single analytical or physics-based mathematical model in practical coating environments where multiple process variables act simultaneously [43].

**Figure 3:**
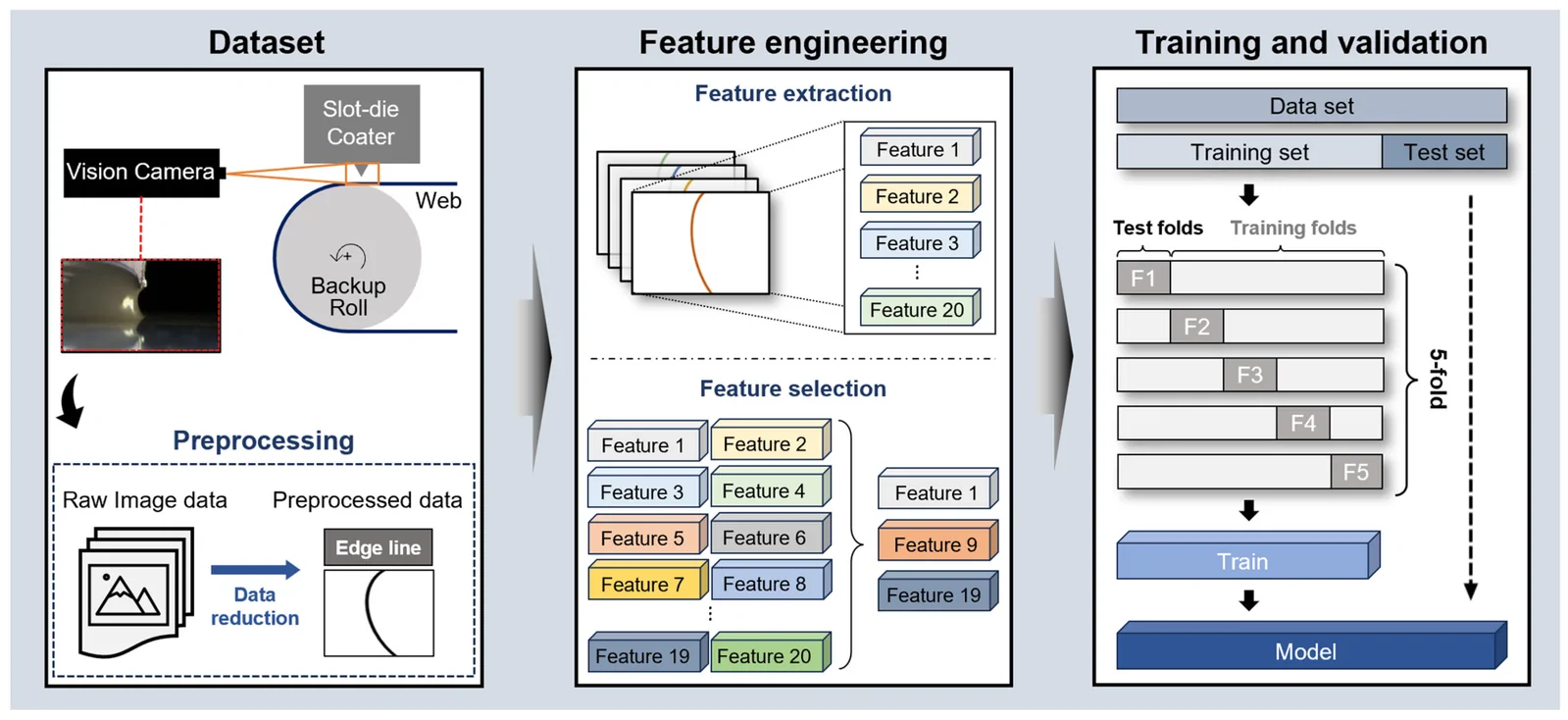
Overall framework of the meniscus vision-based roll eccentricity prediction model.

To address this challenge, this paper presents a machine-learning-based roll eccentricity prediction framework that utilizes meniscus image data. Machine-learning techniques enable the extraction of patterns from multivariate process data and can capture nonlinear relationships and interactions among variables without relying on explicit physical modeling. Consequently, roll eccentricity can be estimated effectively based on the information embedded in meniscus morphology without the need for complex flow analysis. Figure 3 presents the overall framework of the proposed meniscus image-based roll eccentricity prediction method, which consists of three main stages: dataset preparation, feature engineering, and training and validation. First, meniscus images are continuously acquired using a vision camera installed in the slot-die coating system. The acquired raw images are preprocessed to remove unnecessary background information and extract the meniscus boundary required for subsequent analysis. Next, 20 morphological features are extracted from the processed meniscus boundary to quantitatively describe changes in meniscus shape. These features include global geometric characteristics, local contact geometry, and curvature-based characteristics. RF-RFE is then applied to evaluate the relative importance of the extracted features and identify an optimal feature subset by removing less informative features. Finally, the selected features are used as inputs to the regression models for roll eccentricity prediction. The dataset is divided into model-selection data and an independent test set. The model-selection data are used for feature selection, model training, and 5-fold cross-validation, whereas the independent test set is excluded from the model-development process and used only for the final evaluation of prediction performance.

### 3.1. Image Preprocessing

**Figure 4:**
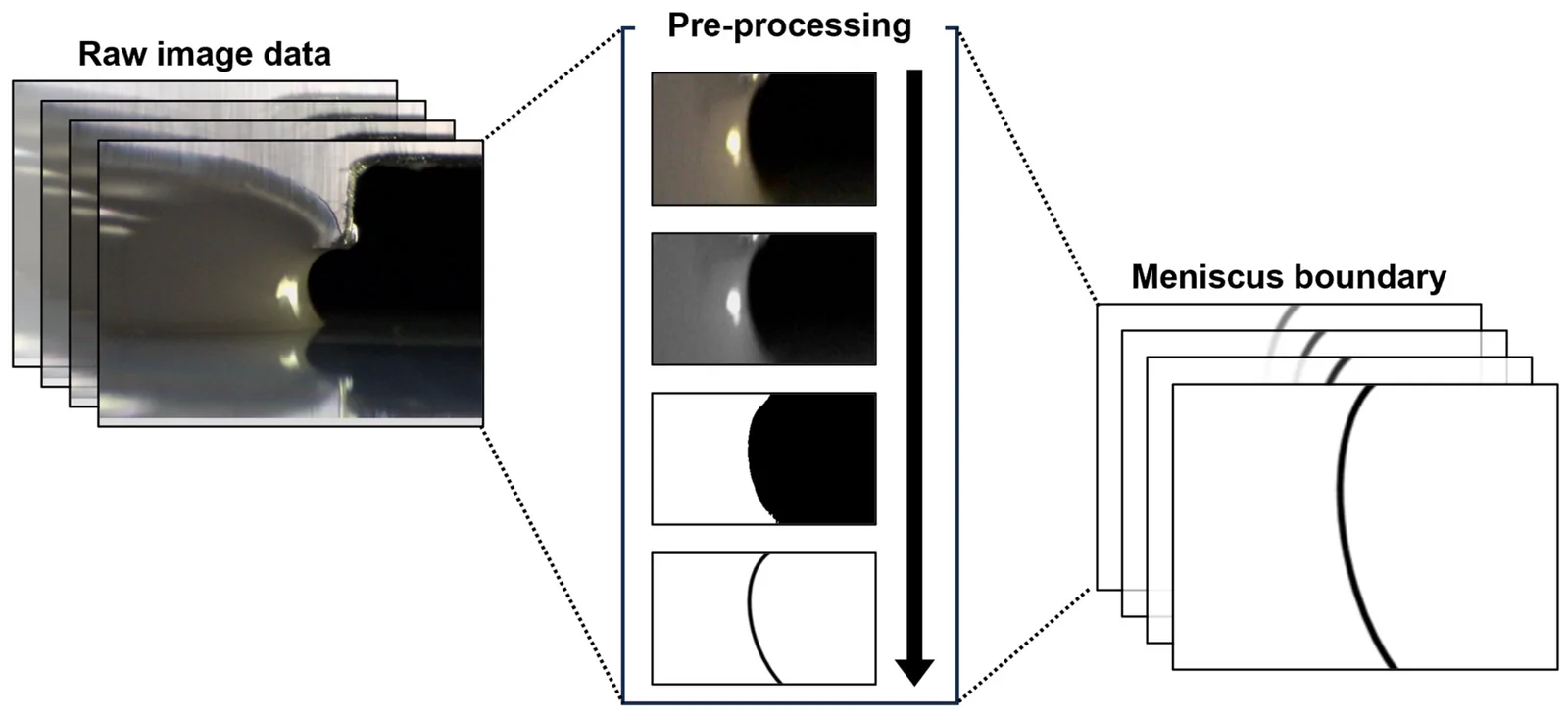
Image preprocessing procedure for meniscus boundary extraction.

Raw images acquired from the R2R slot-die coating system contained background elements, redundant visual information, and pixel-level details with excessive resolution relative to the analysis objective. These characteristics can degrade data-processing efficiency, and therefore, a preprocessing procedure is required to transform raw images into a dataset suitable for model training [44]. Proper preprocessing not only improves prediction accuracy but also enhances training efficiency [45]. In this study, meniscus images were processed through a five-step preprocessing procedure, as illustrated in Figure 4. Figure 4 shows how the continuously acquired raw meniscus image dataset was sequentially converted into meniscus boundary data. The stacked images on the left represent the raw meniscus images acquired during the coating experiment, whereas the subsequent images show representative outputs from each preprocessing step.

Step 1: Meniscus images were continuously acquired using a vision camera installed under predefined processing conditions. The images were captured at intervals of 0.1 s and stored in synchronization with the roll rotational angle information. These continuously acquired images constituted the raw image dataset shown on the left side of Figure 4.

Step 2: From the acquired images, only the region where the meniscus was formed was selected as the region of interest, and cropping was applied to remove unnecessary background information. The cropping region was fixed at the same size for all images to ensure consistency.

Step 3: The cropped images were converted to grayscale to remove color information while retaining only the intensity information. The grayscale pixel values ranged from 0 to 255 and provided the intensity information required for the subsequent binarization process.

Step 4: Based on the grayscale intensity information obtained in Step 3, image binarization was performed to distinguish the meniscus region from the background. The threshold value for the effective separation of the two regions was automatically determined using Otsu’s method [46].

Step 5: Canny edge detection was applied to the binarized images to extract contours corresponding to the boundary between the meniscus and the background [47]. Based on the extracted boundary contours, morphological features were derived to quantitatively characterize geometric variations in the meniscus shape. Thus, each raw image was ultimately converted into a simplified meniscus boundary representation, which was subsequently used to extract the morphological features described in Section 3.2.

### 3.2. Extraction of Morphological Features

**Figure 5:**
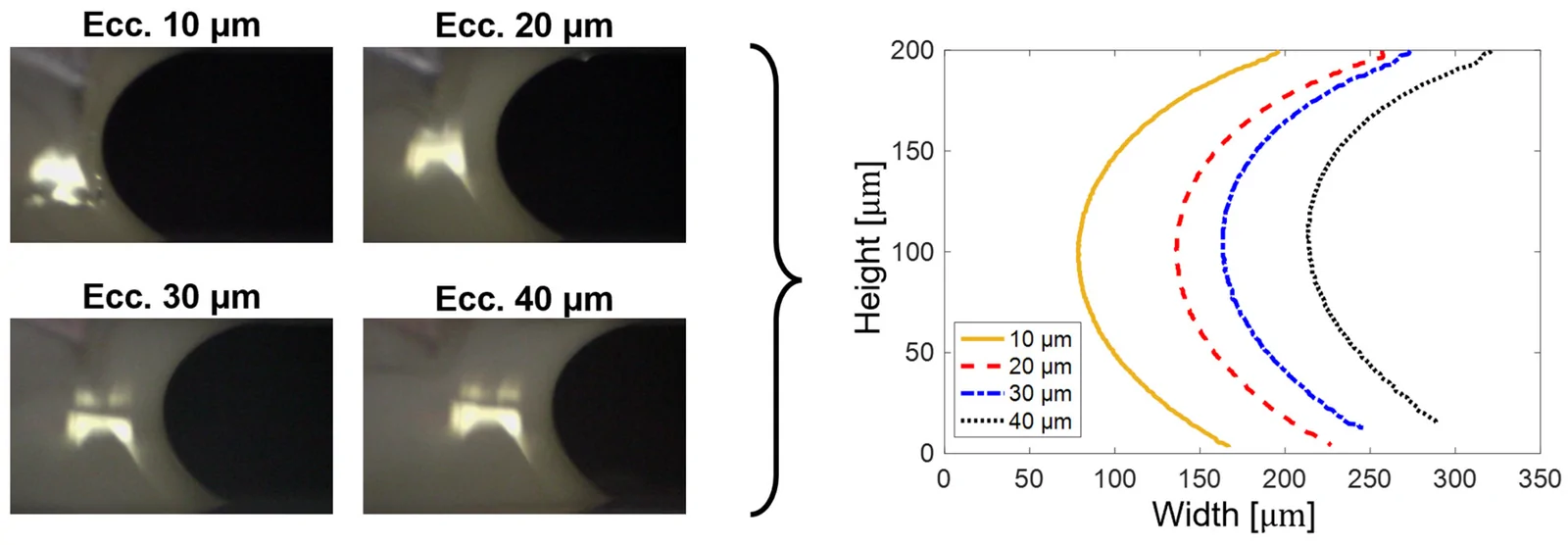
Meniscus images and extracted boundaries under different roll eccentricity conditions.

Figure 5 presents representative meniscus images and corresponding extracted boundaries obtained under different roll eccentricity conditions. Images were acquired for eccentricity values of 10, 20, 30, and 40 μm to examine variations in meniscus shape with respect to eccentricity magnitude, and the meniscus boundaries for all conditions were extracted using an identical preprocessing procedure. The results confirmed that the meniscus shape exhibited a gradual positional shift with an increase in the eccentricity magnitude, while the geometric characteristics of the extracted boundary changed systematically. These shape variations confirm that flow unsteadiness induced by roll eccentricity was reflected in the meniscus boundary, manifesting as consistent and distinguishable patterns under different conditions. Accordingly, geometric changes in the meniscus boundary can serve as meaningful indicators of the presence and magnitude of roll eccentricity in the coating process.

Meniscus images represent unstructured data that do not inherently encode temporal information. Given these data characteristics, statistical variables employed in tension-based prediction models cannot be directly adopted as features for meniscus-based analyses [48]. Therefore, defining a new set of features that can directly capture variations in the meniscus morphology is necessary. Variations in process parameters such as web tension, coating gap, and web speed affect the meniscus flow behavior through different rheological mechanisms [49]. This study defined and extracted a diverse set of morphological features from the meniscus boundary to systematically reflect the effect of these process parameters on the meniscus shape. As summarized in Table 1, 20 features were categorized into three groups: global geometry, local geometry, and curvature-based. Each feature category was designed to capture the distinct rheological mechanisms associated with process-parameter variations. Collectively, these features provide a quantitative representation of the meniscus boundary deformation induced by roll eccentricity, thereby effectively characterizing eccentricity-related process disturbances.

#### 3.2.1. Global Geometry Features

Variations in the coating gap induced by roll eccentricity alter the pressure and velocity distributions within the gap. These changes resulted in variations in the overall height and width of the meniscus. In addition, eccentricity-induced fluctuations in web tension affect the surface condition and wettability of the web, modifying the effective contact area between the coating liquid and substrate. This rheological behavior leads to global geometric changes in the meniscus, including changes in its cross-sectional area, height, and length. Global geometric features were defined to quantitatively characterize the overall contour variation of the meniscus induced by roll eccentricity. These features are summarized as Numbers 1–10 in Table 1 and include representative metrics such as the curve area, curve length, curve height, and curve width.

#### 3.2.2. Local Geometry Features

Fluctuations in web tension caused by roll eccentricity affect contact conditions at the upper and lower interfaces of the meniscus. Localized increases in the web tension increase the surface energy of the web when eccentricity is present. The contact angle of the coating liquid decreases with an increase in surface energy, causing the liquid to spread more extensively over the web surface. Local geometric features are defined to capture these force variations at the liquid–solid interface. These features are summarized as Numbers 11–14 in Table 1 and include the top contact angle, bottom contact angle, top contact length, and bottom contact length, which directly reflect the changes in the interfacial contact behavior induced by eccentricity.

#### 3.2.3. Curvature-Based Features

The meniscus curvature reflects how the interfacial shape responds to variations in process conditions. Fluctuations in the web speed and coating gap induced by roll eccentricity alter flow conditions and pressure equilibrium at the meniscus interface, directly affecting curvature characteristics. An increase in the web speed leads to a higher capillary number, thereby resulting in abrupt changes in the curvature at the meniscus–air interface. Further, variations in the coating gap modify pressure distribution within the coating bead, and these pressure changes induce variations in the meniscus curvature according to the Young–Laplace equation. Pointwise curvature values were calculated at multiple discrete points along the meniscus boundary to quantitatively capture these curvature characteristics. Based on the computed pointwise curvature, geometric statistical measures including the mean, maximum, and standard deviation were extracted and defined as curvature-based features. These features are summarized as Numbers 15–20 in Table 1 and effectively quantify shape variations such as the curvature magnitude and asymmetry, capturing the dynamic deformation behavior of the meniscus.

### 3.3. Feature Selection and Regression Model Development

**Figure 6:**
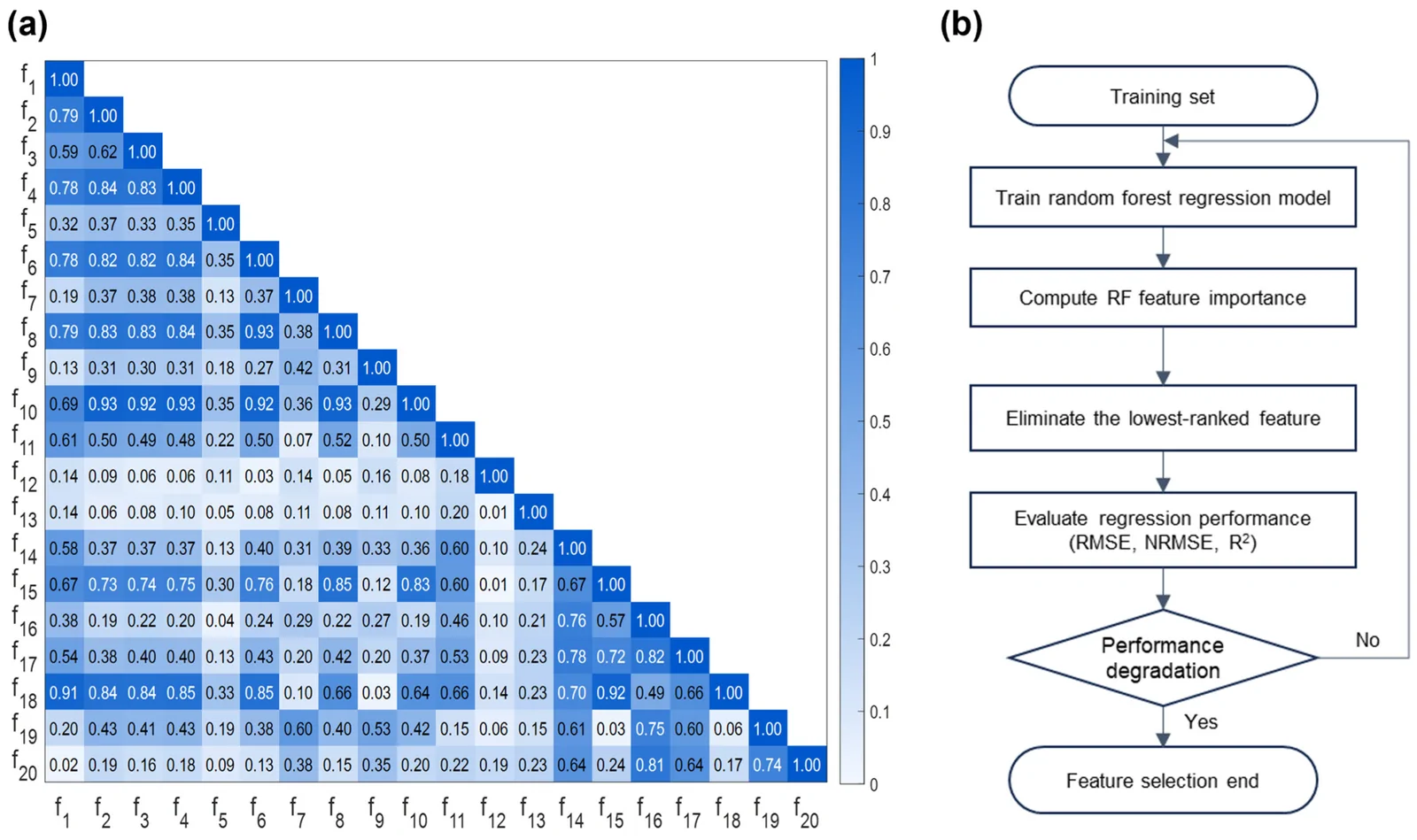
(**a**) Spearman correlation matrix of extracted morphological features. (**b**) Workflow of the RF-RFE method.

The 20 morphological features extracted from the meniscus boundary captured the effects of process parameter variations induced by roll eccentricity from multiple perspectives. A substantial level of interfeature correlation may exist among these features. Figure 6a presents the correlation analysis of 20 features using the Spearman correlation coefficient. For several feature pairs, high correlation coefficients exceeding 0.8 are observed, which indicate potential redundancy among features. Such redundancy can unnecessarily increase the input dimensionality of the model, thereby leading to overfitting and degradation of the generalization performance [50,51,52,53]. Therefore, it is necessary to identify a subset of core features that effectively contribute to eccentricity prediction while maintaining low redundancy.

To address this issue, this study adopts a random forest recursive feature elimination (RF-RFE) method to determine an optimal feature subset. The overall procedure follows the workflow illustrated in Figure 6b. An RF regression model was trained for evaluating the importance of each feature. RF can effectively model nonlinear relationships and capture interactions among features [54], making it suitable for analyzing data characterized by physical interdependencies such as meniscus morphology. Feature importance was quantified using the mean decrease in impurity, which is a widely used metric in RF-based models. This enables the relative contribution of each feature to be evaluated and ranked quantitatively [55]. RFE was subsequently applied based on the derived feature importance ranking; RFE is a wrapper-based feature selection technique that iteratively removes features with the lowest importance, while retraining the model and evaluating its performance at each step [56]. In this study, RFE was performed using the feature importance ranking obtained from the RF model for constructing a compact set of key features most effective for roll eccentricity prediction. The feature rankings and reduced feature subsets obtained through the RF-RFE process are subsequently considered in the analysis of prediction performance as a function of the number of selected features.

### 3.4. Performance Evaluation Metrics

The prediction performance was evaluated using three regression models: RF, eXtreme Gradient Boosting (XGBoost), and support vector regression (SVR). These models represent regression algorithms with different learning characteristics and are selected for verifying the generalization performance of the proposed feature selection method [57,58,59]. For each model, the prediction performance is compared as a function of the number of selected features by applying the same order of feature elimination. The prediction accuracy is evaluated using the RMSE, NRMSE, and R^2^. The mathematical definitions of these metrics are respectively expressed as

(10) $$RMSE=\sqrt{\frac{1}{n}\sum_{i=1}^{n}{\left(y_{i}-{\hat{y}}_{i}\right)}^{2}},$$

(11) $$NRMSE=\frac{1}{\bar{y}}\sqrt{\frac{1}{n}\sum_{i=1}^{n}{\left(y_{i}-{\hat{y}}_{i}\right)}^{2}},$$

(12) $$R^{2}=1-\frac{\sum_{i=1}^{n}{\left(y_{i}-{\hat{y}}_{i}\right)}^{2}}{\sum_{i=1}^{n}{\left(y_{i}-\bar{y}\right)}^{2}},$$

where $y_{i}$ and ${\hat{y}}_{i}$ represent the measured and predicted values of the i-th sample, respectively, and $\bar{y}$ represents the mean of the measured values. The RMSE indicates the absolute magnitude of the prediction error and is sensitive to large errors. The NRMSE is obtained by normalizing the RMSE by the mean of measured values, and it is suitable to compare the relative prediction performance across conditions with different eccentricity magnitudes. R^2^ represents how well the model explains the variability of the measured data, with values closer to 1 indicating superior prediction performance.

## 4. Experimental Setup

### 4.1. Experimental Design and Data Acquisition

**Figure 7:**
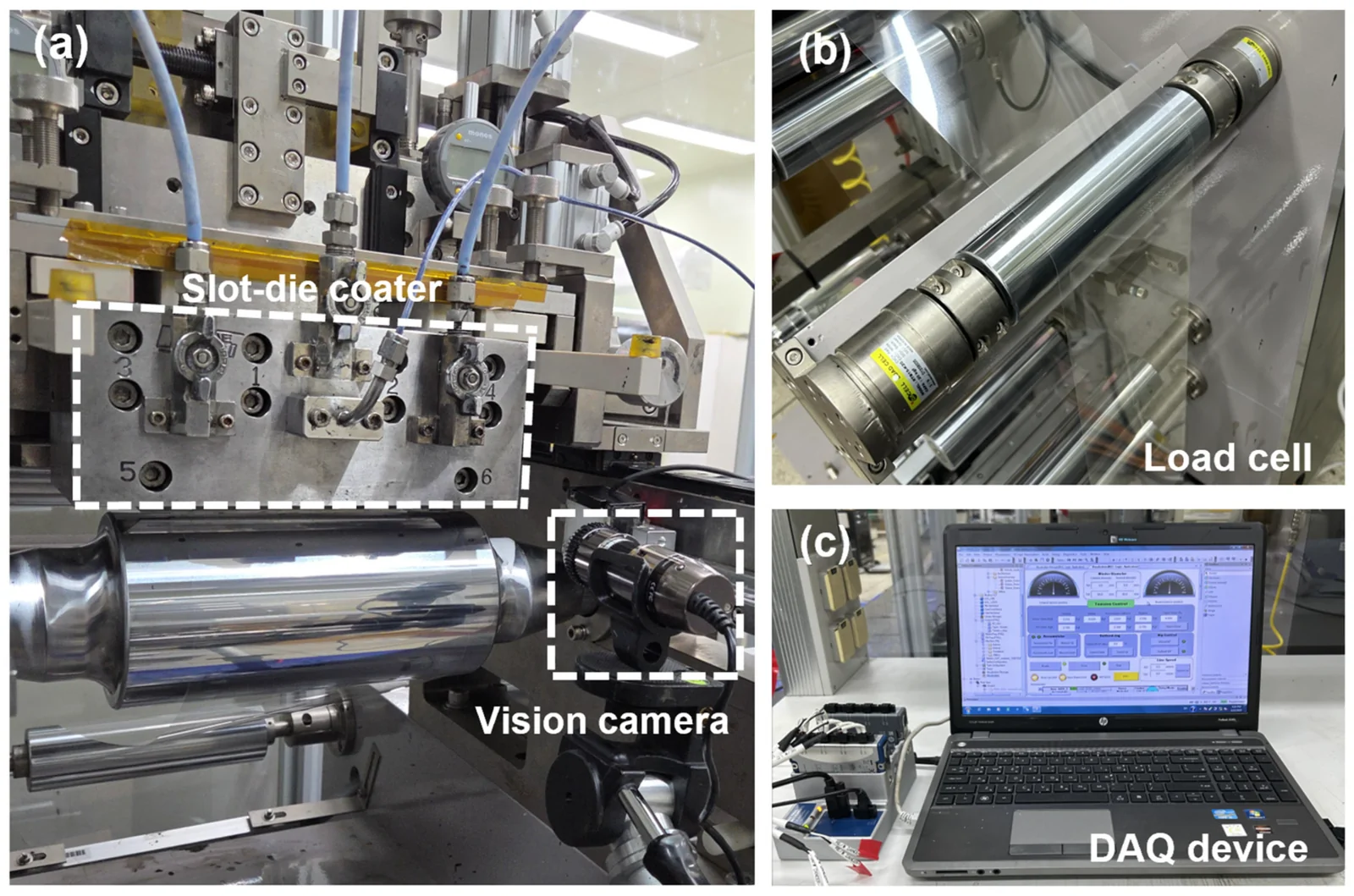
Experimental setup for data acquisition: (**a**) Slot-die coater and vision camera. (**b**) Load cell for tension measurement. (**c**) Tension data acquisition system.

A series of experiments were conducted to analyze the correlation between the meniscus images and roll eccentricity and develop an eccentricity prediction model based on this relationship. An industrial R2R system equipped with a slot-die coating system (Nare Tech, Seoul, Republic of Korea) at Konkuk University, Republic of Korea, was used, as illustrated in Figure 7a. Figure 7a presents the main coating and image-acquisition section, in which the slot-die coater and vision camera are positioned around the backup roll. A vision camera (AM7915MZTL Dino-Lite Edge, Dino-Lite, Seoul, Republic of Korea) was installed at the front-facing position of the backup roll to capture the meniscus shape along the MD accurately. The backup roll angle was measured using an encoder mounted on the roll shaft. The encoder signal was converted into the angular position within one revolution, and each meniscus image was synchronized with the corresponding roll angle. The angular reference was aligned with the coordinate measuring machine (CMM)-measured radial deviation profile. An LED light source was positioned on the backup roll side to ensure consistent illumination.

A load cell (RTB15-K20, DACELL Inc., Cheongju, Republic of Korea) was installed within the web transport span to measure tension fluctuations induced by roll eccentricity, as shown in Figure 7b. The measured tension signals were acquired using a data acquisition unit (NI-9239, National Instruments Corp., Austin, TX, USA), as shown in Figure 7c. The acquired electrical signals were subsequently converted into web tension data for analysis. The electrical signals obtained from the sensor were converted into tension data using a data acquisition system. Subsequently, the resulting tension data were used as reference data for comparing the prediction performance of the proposed meniscus image-based method with conventional tension-based approaches.

The web material used in this study was a polyethylene terephthalate (PET) film (CH34P PET, KOLON Inc., Seoul, Republic of Korea), and a silver nanoparticle ink (JSA101-A, Novacentrix, Austin, TX, USA) was employed as the coating solution. The material properties of the web and coating ink are listed in Table 2.

The applied web tension is set to ~10–25% of the material yield strength to ensure stable web transport and prevent wrinkling [60]. Based on this guideline and considering the mechanical properties of the PET film, the web tension was set to 176.4 N/m in this study. The coating gap was determined to be 200 µm to simultaneously ensure process stability and sufficient sensitivity to roll eccentricity. The web speed and coating flow rate were set to 0.5 m/min and 3 mL/min, respectively. The drying zone temperature was maintained at 90 °C in accordance with the thermal curing requirements of silver nanoparticle ink. The image sampling rate of the vision camera was set to 10 Hz, and approximately 18,000 images were acquired over a total experimental duration of 30 min. The detailed values of the experimental conditions for each test case are summarized in Table 3.

### 4.2. Roll Geometry Measurement

Prior to the experiments, a geometric analysis was conducted for characterizing the cross-sectional shapes of actual backup rolls with different usage histories, i.e., newly manufactured and long-term rolls. Verifying the consistency of the cross-sectional shape along the width direction in advance is necessary because the proposed meniscus image-based prediction model was trained based on a specific roll cross-sectional geometry. To this end, the circularity-based radial profiles of the backup rolls were measured using a coordinate measuring machine (CMM, Model VICTOR101208, Dukin Co., Ltd., Daejeon, Republic of Korea). The CMM had a measurement resolution of 0.05 μm, which was considered when interpreting the measured radial-deviation profiles.

**Figure 8:**
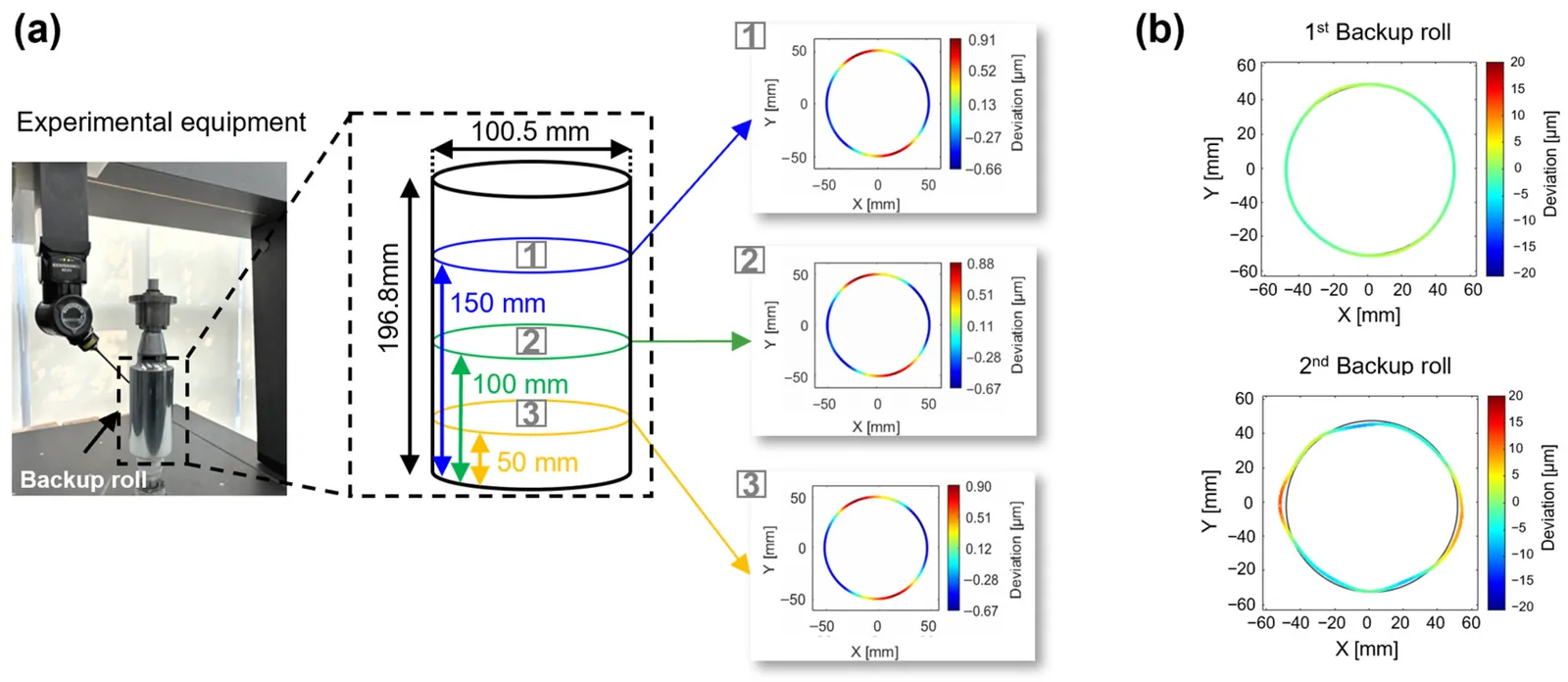
Measurement and analysis of backup roll geometry: (**a**) Roundness profiles at different widthwise positions; (**b**) comparison of cross-sectional roundness between two backup rolls.

As illustrated in Figure 8a, radial profiles at identical angular positions are measured at three widthwise locations (50, 100, and 150 mm) from the roll edge for each backup roll to examine the consistency of the cross-sectional geometry along the width direction. A comparison of radial profiles obtained at three widthwise locations revealed that radius differences were less than 0.1 µm in all cases. This result confirms that the cross-sectional geometry of both backup rolls remained nearly identical regardless of the widthwise position, confirming that the cross-sectional shape measured at a single width location can be considered representative of the entire roll.

However, the circumferential radius variation can differ significantly depending on the wear and deformation states of the roll even with a consistent cross-sectional geometry along the width direction. Circularity measurements were performed for both backup rolls at the same width (50 mm) to investigate this effect, and their circumferential cross-sectional profiles were compared, as shown in Figure 8b. The measurement results confirm that the first backup roll (new roll) exhibits negligible wear and deformation, with an overall radius variation maintained within ±1 µm. In contrast, the second backup roll (long-term used roll) shows pronounced eccentricity caused by wear and deformation, with the overall radius variation increasing substantially to approximately ±20 µm. These results indicate that the circumferential radius variation, i.e., deterioration in circularity, can increase significantly depending on the usage history and wear condition of the roll while the widthwise cross-sectional geometry of a backup roll can remain relatively uniform. Based on this preliminary geometric analysis, the first backup roll with a verified geometric consistency was selected as the reference roll in this study, and subsequently, experimental cases were constructed by introducing artificial eccentricity.

### 4.3. Construction of the Roll Eccentricity Test Case

**Figure 9:**
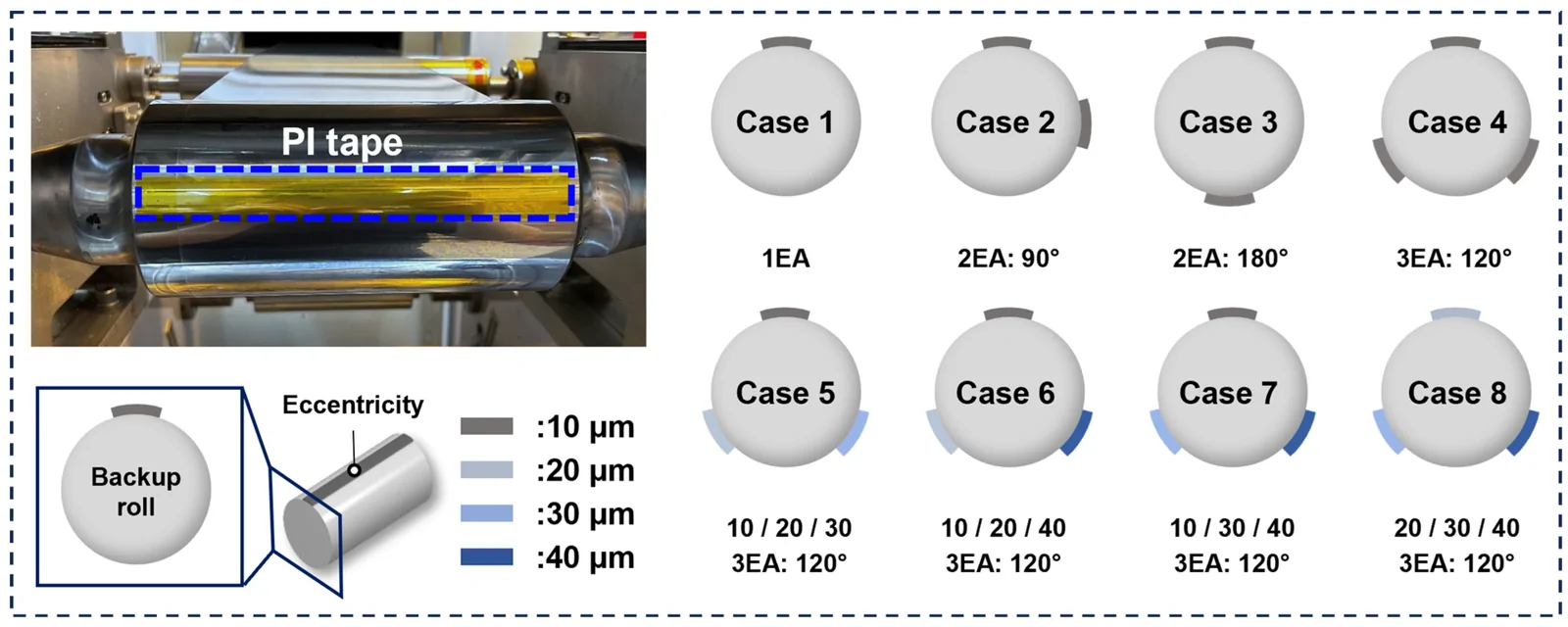
Construction of eccentric backup rolls for experimental validation.

A series of experimental cases was constructed by introducing artificial eccentricity to the first backup roll for validating the performance of the roll eccentricity prediction model. The geometric consistency of this roll was verified in the previous section. The configurations of the experimental cases are illustrated in Figure 9. Artificial eccentricity is implemented by attaching polyimide tape with a thickness of 10 µm to the surface of the backup roll located beneath the slot-die coater. A total of 8 eccentricity cases were designed, and the detailed conditions for each case are summarized in Table 4. Cases 1–4 were configured using the same eccentricity magnitude, whereas varying the number and angular positions of the eccentricities. This design enables an analysis of how the number of eccentricities present on a single roll affects prediction accuracy. In Cases 5–8, three eccentricities were attached at 120° intervals, and the magnitude of each eccentricity varied. This configuration enables evaluating the effect of the eccentricity magnitude distribution on the prediction performance when the eccentricities are located at identical angular positions. All eccentricities were attached under identical conditions using predefined reference lines and angular guides to ensure consistent positional alignment and experimental reproducibility.

After constructing each eccentricity case, the induced radial-deviation profiles were verified using the same CMM measurement procedure described in Section 4.2. Measurements were conducted at three widthwise positions (50, 100, and 150 mm), and each position was measured three times. Across all eccentricity cases, the maximum difference among the three repeated measurements at the same position was within 0.05 μm, while the maximum difference among the three widthwise positions was below 0.10 μm. In addition, the maximum absolute deviation between the nominal eccentricity defined by the total tape thickness and the CMM-measured eccentricity was within 0.20 μm. These results confirmed the repeatability and consistency of the constructed eccentricity conditions. The CMM-measured radial-deviation profiles were subsequently used as the reference values for model evaluation. The measurement characteristics are summarized in Table 5. These eccentricity configurations were designed for systematically evaluating the effect of variations in eccentricity magnitude and distribution on the prediction performance of the proposed meniscus image-based model.

## 5. Results and Discussion

The performance of the proposed meniscus image-based eccentricity prediction framework is validated using experimental results. A feature importance analysis and comparative evaluation of the prediction models were conducted using representative eccentricity cases. Based on this analysis, a prediction model and feature subset commonly applicable to all cases were selected.

### 5.1. Selection of the Prediction Model and Optimal Feature Subset

**Figure 10:**
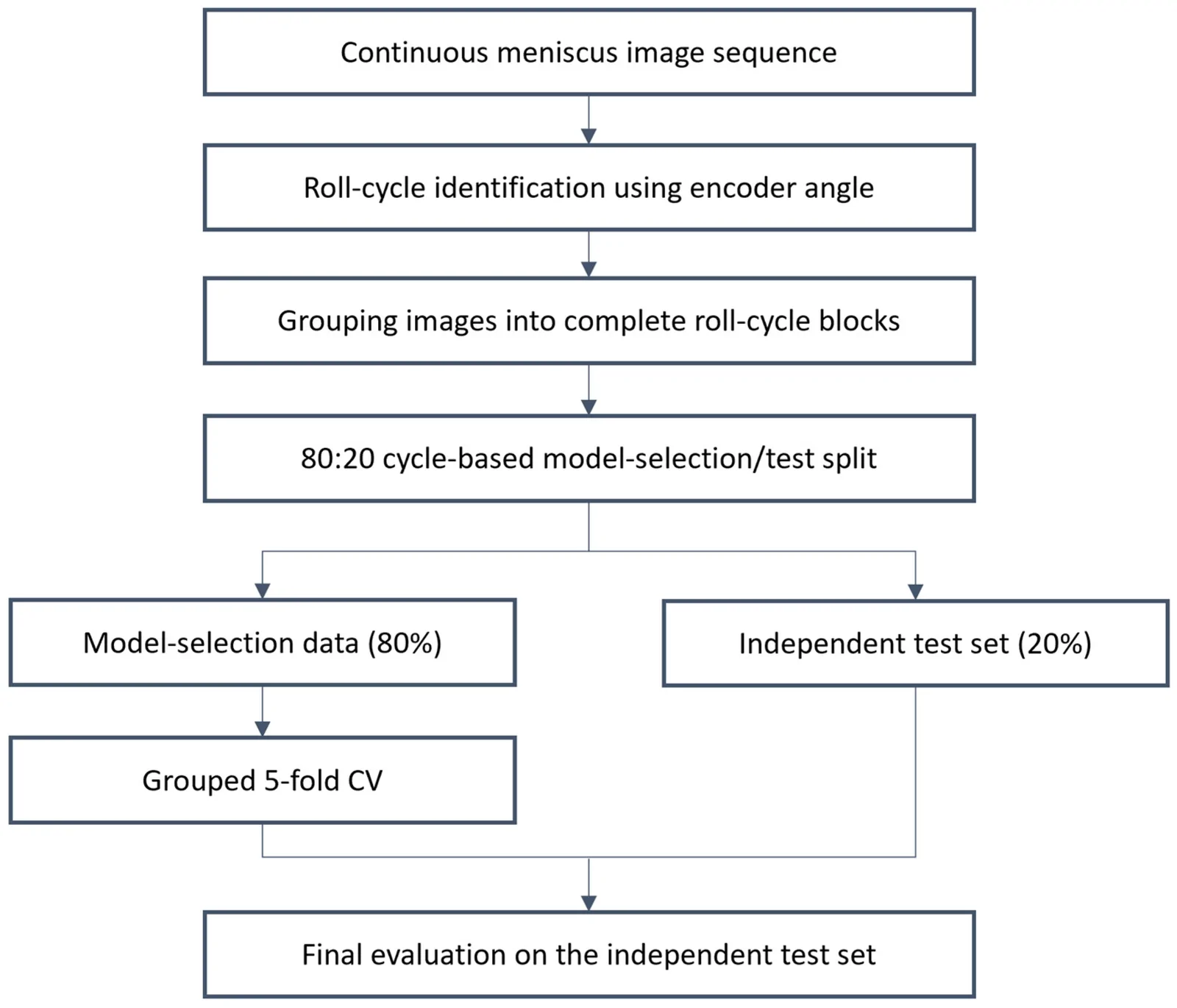
Roll-cycle-based data partitioning and cross-validation procedure.

An analysis was performed using a balanced dataset consisting of 100 meniscus images for each eccentricity case to select an appropriate prediction model and feature subset. To ensure diverse representation, the images were uniformly sampled over the roll rotation cycle rather than selected from consecutive frames. Figure 10 presents the roll-cycle-based data partitioning and cross-validation procedure used in this study. First, the continuous meniscus image sequence was assigned to individual roll cycles using the encoder-angle information, and all images belonging to the same roll revolution were grouped into complete roll-cycle blocks. These cycle blocks were then divided into model-selection data and an independent test set at an 80:20 ratio. Within the model-selection data, grouped 5-fold cross-validation was performed while keeping each roll-cycle block within a single fold. In each iteration, four folds were used for training and the remaining fold was used for validation. Thus, images from the same roll cycle were not shared across the model-selection and test sets or between the training and validation folds. The model-selection data were used for RF-RFE, model training, and cross-validation, whereas the independent test set was used solely for the final performance evaluation. The resulting data partitioning strategy is summarized in Table 6.

**Figure 11:**
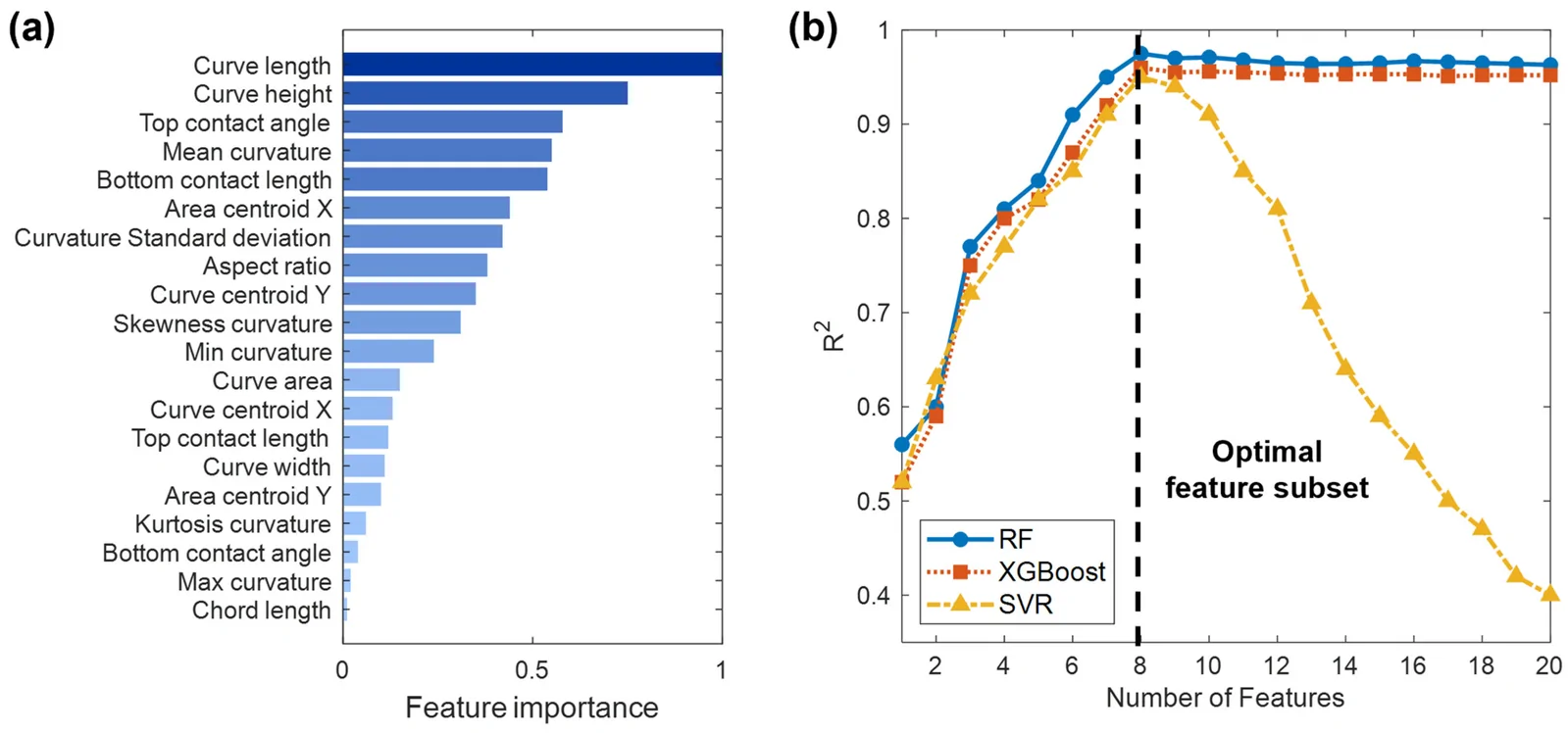
(**a**) RF feature importance ranking. (**b**) Prediction performance comparison among RF, XGBoost, and SVR models.

Figure 11a shows the normalized feature importance rankings obtained by applying the RF model to the training data. Among global geometric features, the curve length and curve height exhibited relatively high importance, suggesting that the overall meniscus shape reflected coating gap variations induced by eccentricity. Among local geometry features, the top and bottom contact angles showed comparatively high importance, indicating the significant role of interfacial contact behavior in eccentricity prediction. Among curvature-based features, the curvature mean exhibited the highest importance. These results indicate that accurate eccentricity prediction requires the combined consideration of global deformation, local contact behavior, and curvature variation.

Based on the feature importance ranking, RFE was applied to compare prediction performance across different feature subset sizes. During each RFE step, the RF hyperparameters were kept fixed, and the least important feature was sequentially removed based on the mean decrease in impurity. The number of selected features was not predetermined. Instead, each feature subset was evaluated using the validation data, and the subset with the highest average validation R^2^ was selected as the optimal feature subset. The independent test set was not used during feature ranking, feature elimination, or optimal feature subset selection.

For fair model comparison, RF, XGBoost, and SVR were trained and validated using the same data partitioning strategy. The main hyperparameter settings used for each model are summarized in Table 7. To obtain a stable estimate of generalization performance, each model was trained and validated using 5-fold cross-validation [61,62]. Figure 11b presents the average validation R^2^ values according to the number of selected features. The prediction performance reached its maximum when eight features were used. In this region, the RF model achieved the highest validation R^2^ with stable performance across folds. Accordingly, RF was selected as the final prediction model, and the eight-feature subset was defined as the optimal feature combination. The selected optimal feature set is listed in Table 8.

### 5.2. Performance Evaluation Across Multiple Eccentricity Cases

**Figure 12:**
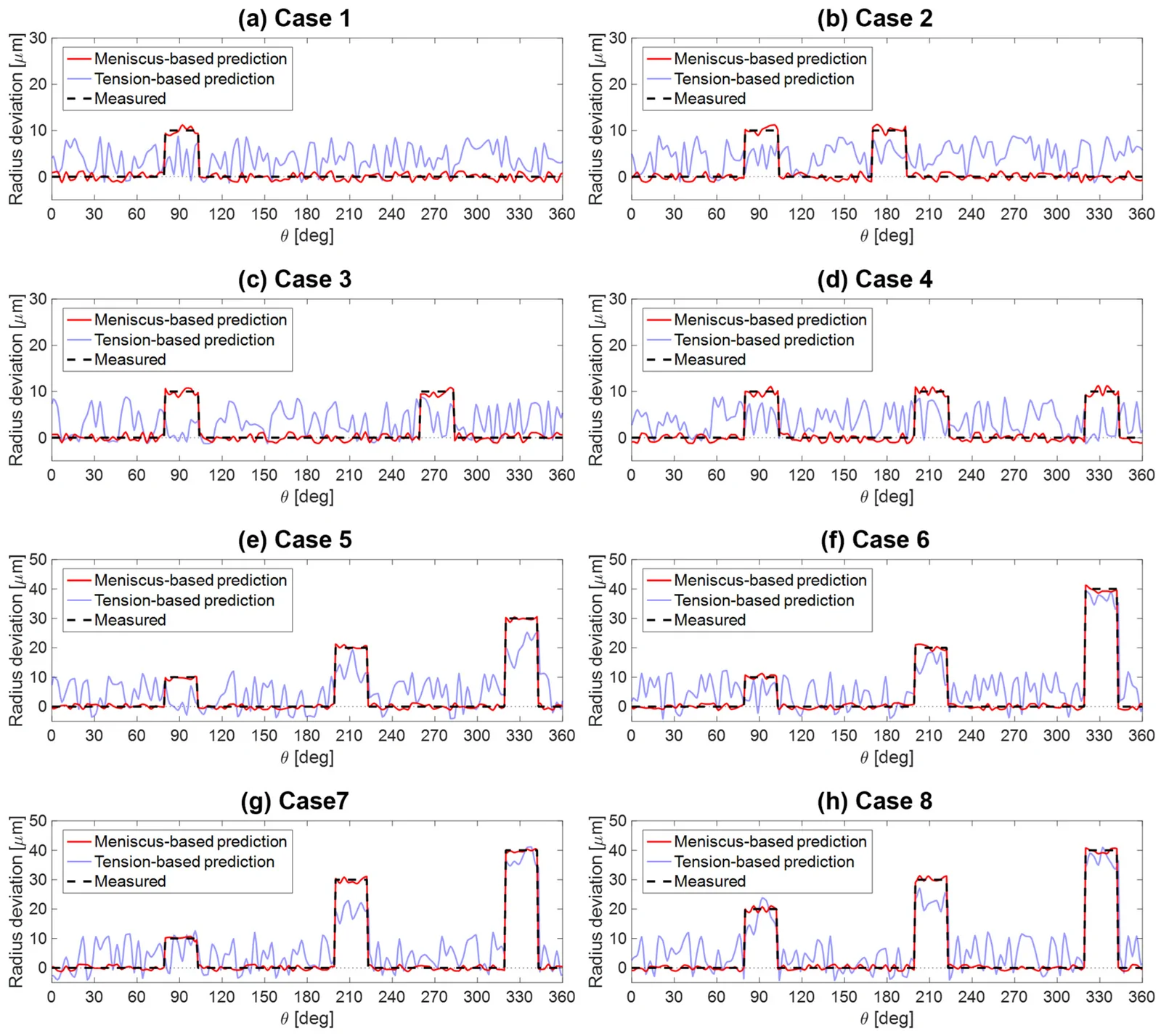
Measured and predicted roll radius deviation results of all eccentricity cases (**a**–**h**): Comparison of measured and predicted roll radius deviations for Cases 1–8, respectively.

The RF-based prediction model with the optimal feature subset was applied uniformly to all eccentricity cases to compare prediction performance. Prediction results obtained from the meniscus image-based and tension signal-based methods were analyzed along with the measured radial deviation. Figure 12 compares the measured radial deviation as a function of the roll rotational angle with predictions obtained from the meniscus image- and tension signal-based models for each eccentricity case. For all eccentricity cases, the meniscus-based predictions exhibited trends that closely matched the measured curves and accurately reproduced the location and magnitude of eccentricity. Even in regions with no eccentricity, the prediction fluctuation remained small, enabling local variations in the radial deviation to be clearly distinguished. In contrast, tension-based predictions exhibited trends that differed from the measured curves. For the fine eccentricity conditions in Cases 1–4, where the eccentricity magnitude is on the order of 10 µm, the tension fluctuation components induced by eccentricity were relatively small, resulting in insufficient SNR compared to process noise. Consequently, the tension-based prediction model failed to clearly distinguish the structural variations induced by roll eccentricity. Thus, the discrimination between regions with and without eccentricity was limited in tension-based predictions.

**Figure 13:**
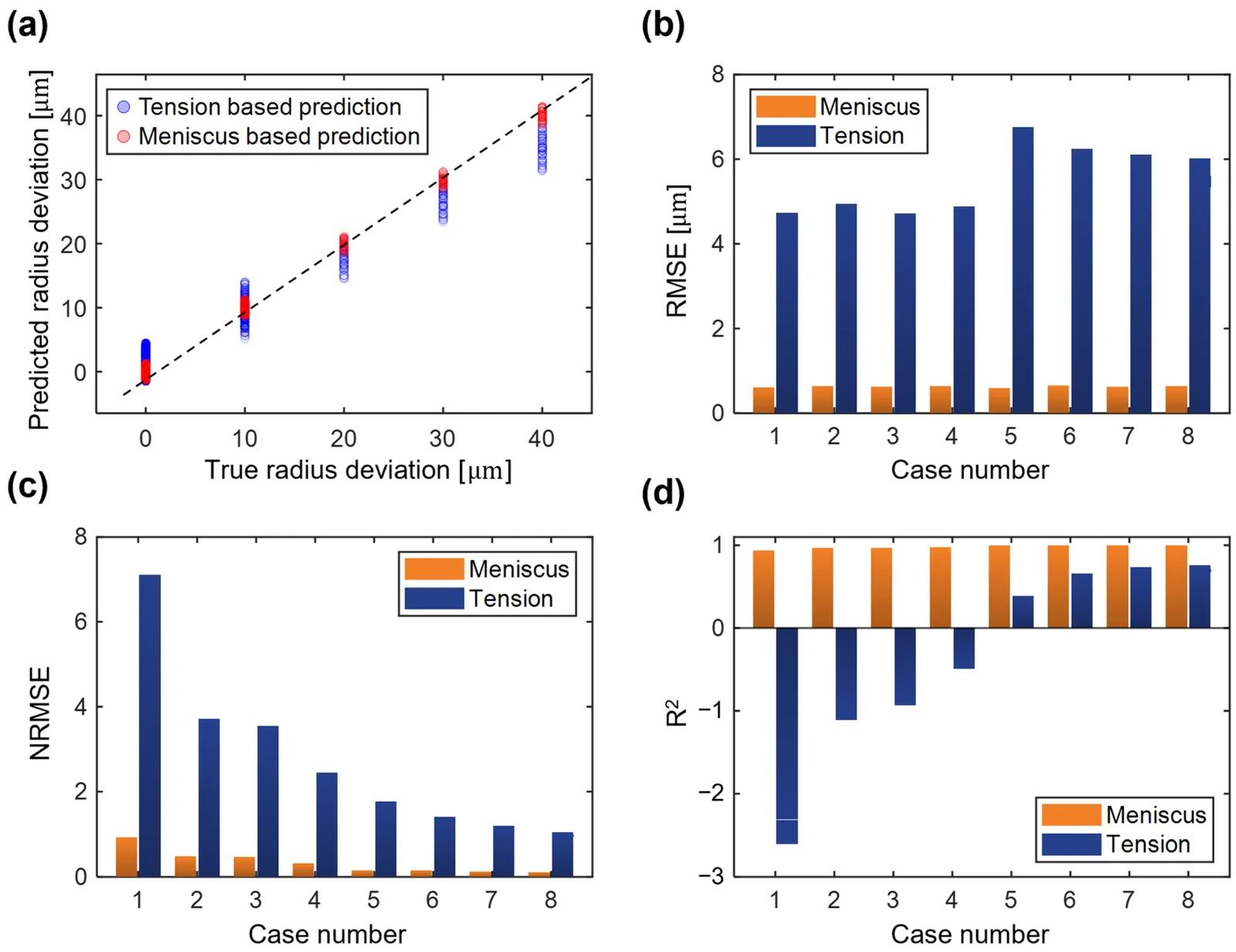
Quantitative performance comparison between meniscus-based and tension-based prediction methods: (**a**) Predicted versus measured radius deviation with the 1:1 reference line, (**b**) RMSE, (**c**) NRMSE, (**d**) R^2^.

The performance metrics for radial deviation are presented in Figure 13 and Table 9. These performance metrics were used to quantitatively evaluate the roll eccentricity prediction capability of the proposed model. Figure 13a shows a scatter plot illustrating the relationship between the measured radial deviation and the predicted values across all eccentricity cases. The meniscus-based predictions are densely distributed around the diagonal line y=x, demonstrating a high level of agreement between predicted and measured values. In contrast, the tension-based predictions exhibit a much wider dispersion in the scatter plot, confirming relatively larger prediction errors. Figure 13b presents a comparison of the RMSE for each eccentricity case. The RMSE of the meniscus-based prediction remains consistently low, with an average value of 0.627 μm, and shows stable error characteristics with minimal variation regardless of changes in the number or magnitude of eccentricities. By contrast, the tension-based prediction yields a significantly larger average RMSE of 5.549 μm, indicating substantially inferior accuracy compared to the meniscus-based approach. Figure 13c compares the NRMSE for each eccentricity case. The meniscus-based predictions exhibited NRMSE values ranging from 0.111 to 0.925, with an average value of 0.342 across the eight eccentricity cases. The meniscus-based predictions consistently maintain lower NRMSE values than the tension-based predictions across all cases. The tension-based approach exhibits relatively large errors under fine eccentricity conditions. However, as the average eccentricity magnitude increases from Case 5 to Case 8, the NRMSE of the tension-based prediction decreases sharply. This trend indicates that tension signals have limited capability in identifying fine eccentricity and can only partially reflect radial deviation when the eccentricity magnitude becomes sufficiently large. These results quantitatively demonstrate that the meniscus-based prediction maintains consistent accuracy regardless of eccentricity number and magnitude, whereas the tension-based prediction exhibits strong dependence on eccentricity magnitude. Figure 13d compares R^2^ for each eccentricity case. The meniscus-based predictions maintain high R^2^ values exceeding 0.93 for all cases and approach values close to 0.99 under conditions with larger eccentricity magnitudes. This indicates that the proposed model explains most of the variability observed in the measured data. In contrast, the tension-based predictions yield negative R^2^ values in several cases, implying that their explanatory power is even lower than that of a simple mean-based model under those conditions. These results confirm that the reliability of tension-based prediction is severely limited, particularly under fine eccentricity conditions.

It should be noted that the present model was developed and evaluated under fixed process conditions, including a web speed of 0.5 m/min, a coating gap of 200 μm, a web tension of 176.4 N/m, and an ink viscosity of 7.7 cP. Since changes in these operating conditions can independently affect meniscus morphology, the relationship between the extracted morphological features and roll eccentricity may also vary. Therefore, the prediction performance of the proposed model has been validated only under the operating conditions investigated in this study, and its generalization to other process conditions requires further experimental validation.

## 6. Conclusions

In this study, a machine-learning-based backup-roll eccentricity prediction model was proposed and experimentally validated, motivated by the observation that the meniscus shape formed in the R2R slot-die coating process responds sensitively to backup-roll eccentricity. Morphological features were extracted from the meniscus boundaries, and an optimal feature subset was derived using RF-RFE. A comparative evaluation of three algorithms (RF, XGBoost, and SVR) demonstrated that the RF model exhibited superior and stable performance, which was therefore selected. Based on the adopted algorithm and feature combination, the final model was constructed and applied to all eccentricity test cases. The resulting average RMSE, NRMSE, and R^2^ values were approximately 0.63 μm, 0.34, and 0.98, respectively, demonstrating consistently improved performance across all metrics compared to the conventional tension-based prediction model. These results indicate that the meniscus-based approach effectively complements the limitations of tension-based prediction and enables more accurate and practical estimation of backup roll eccentricity. In addition, the proposed model confirms that complex process states can be effectively diagnosed in a data-driven manner based on the intrinsic information embedded in meniscus morphology without relying on detailed physical modeling. Furthermore, it can be implemented with the addition of a relatively simple vision sensor, which makes it highly feasible for applications in industrial coating processes.

In future work, the experimental dataset will be extended to include multiple operating conditions, such as different web speeds, coating gaps, web tension levels, and coating-material properties, to evaluate the robustness and generalization capability of the proposed method. Model retraining or calibration strategies will also be investigated to accommodate variations in operating conditions. In addition, deep learning–based feature learning will be explored to capture nonlinear meniscus shape variations more directly. Adaptive learning strategies will also be investigated to accommodate roll wear and system changes over time and extend the proposed framework toward reliable long-term backup roll diagnosis and predictive maintenance in industrial R2R coating processes.

## Figures and Tables

**Table 1:** Definition of extracted meniscus morphological features.

No.	Feature	Definition
1	Curve area	Internal area enclosed by the meniscus curve
2	Curve length	Total length of the meniscus curve
3	Curve height	Maximum vertical height of the meniscus curve
4	Curve width	Maximum horizontal width of the meniscus curve
5	Aspect ratio	Maximum height-to-width ratio of the meniscus curve
6	Chord length	Distance between the two endpoints of the meniscus curve
7	Curve centroid point X	X-coordinate of the curve centroid
8	Curve centroid point Y	Y-coordinate of the curve centroid
9	Area centroid point X	X-coordinate of the centroid of the enclosed area
10	Area centroid point Y	Y-coordinate of the centroid of the enclosed area
11	Top contact length	Length of the upper contact line
12	Bottom contact length	Length of the lower contact line
13	Top contact angle	Contact angle at the upper boundary
14	Bottom contact angle	Contact angle at the lower boundary
15	Curvature mean	Mean curvature along the meniscus boundary
16	Curvature standard deviation	Standard deviation of curvature along the meniscus boundary
17	Curvature max	Maximum curvature along the meniscus boundary
18	Curvature min	Minimum curvature along the meniscus boundary
19	Curvature skewness	Skewness of curvature along the meniscus boundary
20	Curvature kurtosis	Kurtosis of curvature along the meniscus boundary

**Table 2:** Material properties of the web and coating ink.

**Web: PET Film (CH34P)**		
**Parameter**	**Unit**	**Value**
Thickness	μm	75
Width	mm	150
Young’s modulus	GPa	3.19
Yield stress	MPa	109.7
Poisson’s ratio	-	0.37
**Ink: Silver nano ink (JS-A101A)**		
**Parameter**	**Unit**	**Value**
Ink viscosity	cP	7.7
Surface tension	mN/m	19
Weight percent	%	40

**Table 3:** Experimental conditions for the slot-die coating process.

Parameter	Unit	Value
Coating gap	μm	200
Web speed	m/min	0.5
Flow rate	mL/min	3
Operating tension	N/m	176.4
Drying temperature	°C	90

**Table 4:** Roll eccentricity test cases used for experimental validation.

Case No.	Eccentricity [EA]	Thickness [µm]	Position [°]
1	1	10	-
2	2	10/10	90°
3	2	10/10	180°
4	3	10/10/10	120°
5	3	10/20/30	120°
6	3	10/20/40	120°
7	3	10/30/40	120°
8	3	20/30/40	120°

**Table 5:** CMM measurement repeatability and consistency of the eccentricity cases.

Measurement Characteristic	Value
CMM resolution	0.05 μm
Widthwise measurement positions	50, 100, 150 mm
Repeated measurements per position	3
Repeated-measurement variation	≤0.05 μm
Widthwise variation	<0.10 μm
Nominal–measured deviation	±0.20 μm

**Table 6:** Dataset partitioning strategy for model development and evaluation.

Dataset	Samples Per Condition	Total Samples
Model-selection data	80	640
Independent test set	20	160
Training fold	64	512
Validation fold	16	128

**Table 7:** Hyperparameter settings of the regression models.

Model	Hyperparameter	Value
RF	Number of trees	500
RF	Maximum tree depth	none
RF	Max features	sqrt
RF	Minimum samples per leaf	1
XGBoost	Number of estimators	300
XGBoost	Learning rate	0.05
XGBoost	Maximum tree depth	5
SVR	Kernel function	RBF
SVR	C	10
SVR	ϵ	0.1
SVR	γ	scale

**Table 8:** Optimal feature subset selected for roll eccentricity prediction.

Rank	Feature	Category
1	Curve length	Global geometry features
2	Curve height	Global geometry features
3	Top contact angle	Local geometry features
4	Curvature mean	Curvature-based features
5	Bottom contact angle	Local geometry features
6	Area centroid X	Global geometry features
7	Curvature standard deviation	Curvature-based features
8	Aspect ratio	Global geometry features

**Table 9:** Quantitative comparison of eccentricity prediction performance using meniscus- and tension-based prediction methods.

Case No.	RMSE		NRMSE		R^2^	
	Meniscus	Tension	Meniscus	Tension	Meniscus	Tension
1	0.615	4.729	0.925	7.114	0.939	−2.604
2	0.638	4.938	0.479	3.714	0.965	−1.115
3	0.621	4.724	0.467	3.553	0.967	−0.936
4	0.645	4.879	0.324	2.446	0.974	−0.491
5	0.589	6.765	0.154	1.770	0.995	0.387
6	0.651	6.236	0.153	1.398	0.996	0.659
7	0.623	6.112	0.122	1.199	0.997	0.733
8	0.634	6.013	0.111	1.049	0.997	0.762

## Data Availability

Data is contained within the article.
